# Field measurements of turbulent mixing south of the Lombok Strait, Indonesia

**DOI:** 10.1186/s40562-024-00349-3

**Published:** 2024-08-14

**Authors:** R. Dwi Susanto, Zexun Wei, Priyadi Dwi Santoso, Guanlin Wang, Muhammad Fadli, Shujiang Li, Teguh Agustiadi, Tengfei Xu, Bayu Priyono, Ying Li, Guohong Fang

**Affiliations:** 1 Department of Atmospheric and Oceanic Science, University of Maryland, College Park, MD 20742 USA; 2 Marine-Estuarine and Environmental Sciences, University of Maryland, College Park, MD 20742 USA; 3 First Institute of Oceanography, and Key Laboratory of Marine Science and Numerical Modeling, Ministry of Natural Resources, Qingdao, People’s Republic of China; 4 Research Center for Deep Sea, National Research and Innovation Agency, Jakarta, Indonesia; 5 Research Center for Oceanography, National Research and Innovation Agency, Jakarta, Indonesia

**Keywords:** Lombok strait, Tidal mixing, Indonesian throughflow, Microstructure profilers, Dissipation rate, Diapycnal diffusivity, Internal tides

## Abstract

The Indonesian seas, with their complex passages and vigorous mixing, constitute the only route and are critical in regulating Pacific–Indian Ocean interchange, air–sea interaction, and global climate events. Previous research employing remote sensing and numerical simulations strongly suggested that this mixing is tidally driven and localized in narrow channels and straits, with only a few direct observations to validate it. The current study offers the first comprehensive temporal microstructure observations in the south of Lombok Strait with a radius of 0.05° and centered on 115.54^o^E and 9.02^o^S. Fifteen days of tidal mixing observations measured potential temperature and density, salinity, and turbulent energy dissipation rate. The results revealed significant mixing and verified the remotely sensed technique. The south Lombok temporal and depth averaged of the turbulent kinetic energy dissipation rate, and the diapycnal diffusivity from 20 to 250 m are $\varepsilon$ = 4.15 ± 15.9) × 10^–6^ W kg^–1^ and $K\rho$= (1.44 ± 10.7) × 10^–2^ m^2^s^–1^, respectively. This $K\rho$ is up to 10^4^ times larger than the Banda Sea [$K\rho$ = (9.2 ± 0.55) × 10^–6^ m^2^s^–1^] (Alford et al. Geophys Res Lett 26:2741–2744, 1999) or the “open ocean” $K\rho$= 0.03 × 10^–4^ m^2^s^−1^ within 2° of the equator to (0.4–0.5) × 10^–4^ m^2^s^−1^ at 50°–70° (Kunze et al. J Phys Oceanogr 36:1553–1576, 2006). Therefore, nonlinear interactions between internal tides, tidally induced mixing, and ITF plays a critical role regulating water mass transformation and have strong implications to longer-term variations and change of Pacific–Indian Ocean water circulation and climate.

**Supplementary Information:**

The online version contains supplementary material available at 10.1186/s40562-024-00349-3.

## Introduction

**Fig. 1:**
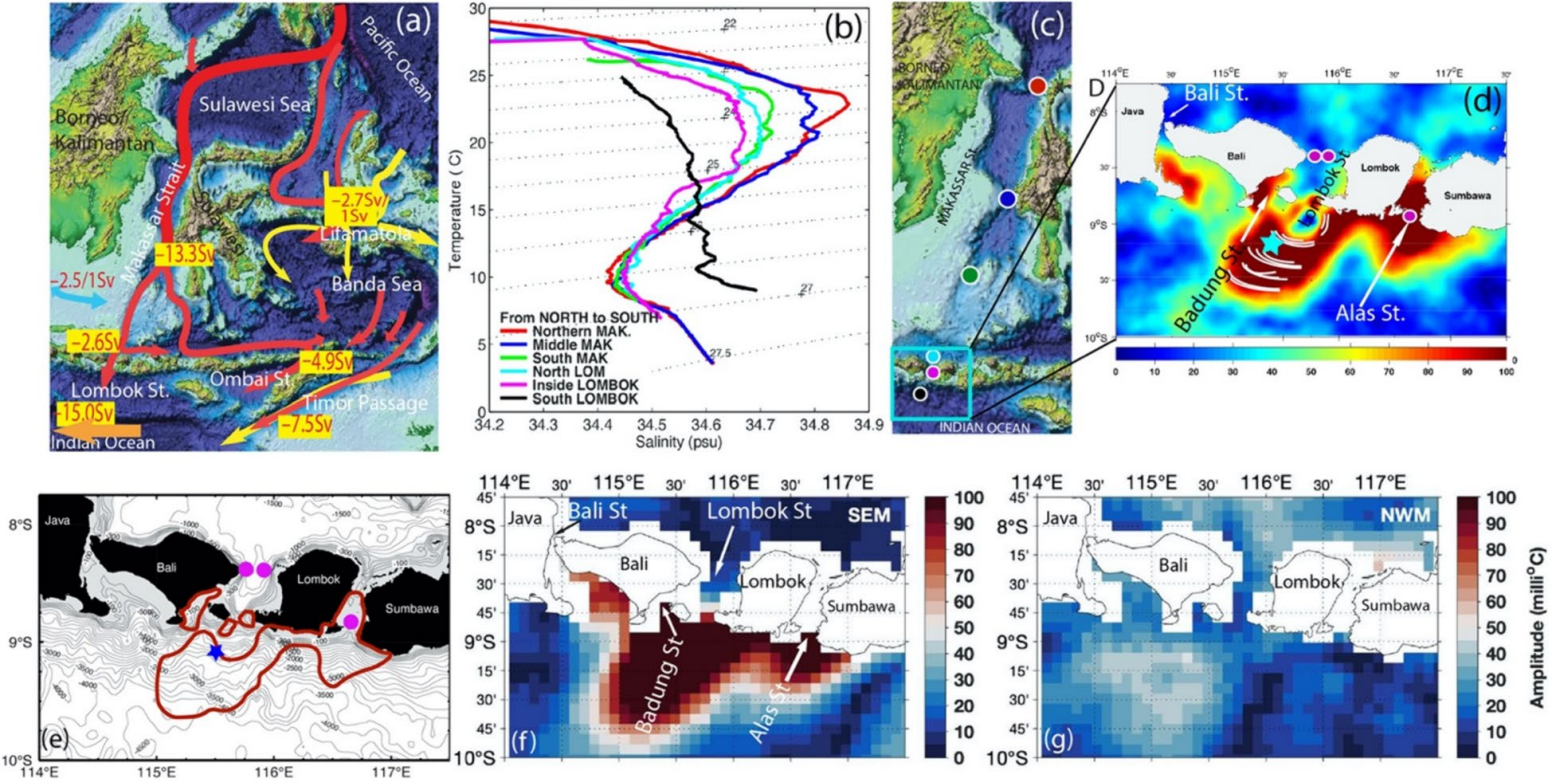
**a** Map of the main Indonesian throughflow (ITF) pathways in the eastern part of Indonesia from the Pacific into the Indian Ocean. b Temperature–salinity (*TS)* diagram of the evolution of water masses from north of Makassar Strait through Lombok Strait into the Indian Ocean taken during the TRIUMPH (ThRoughflow Indonesian seas, Upwelling, and Mixing PHysics) cruise in December 2019, revealing strong mixing in Makassar and Lombok Straits for potential densities σ_θ_ = 23–27 kg m^−3^ (modified from Susanto and Ray 2022). **c** Map of the Makassar and Lombok Straits showing the location of the stations used in (**b**). **d** Estimates of fortnightly (MS_*f*_) SST amplitude in milli^o^C (Susanto and Ray 2022; Ray and Susanto 2016). White lines in the SST anomaly south of Lombok Strait coincide with soliton signatures observed by SAR satellites (Susanto et al. 2005; Matthews et al. 2011). The high SST amplitudes in d are consistent with tidal mixing which we hypothesize is responsible for the abrupt changes in the *T–S* diagram from inside to the south of Lombok Strait indicate strong mixing as waters pass over the sills at the southern end of Lombok Strait seen in (**b**). **e** Map of the Lombok Strait showing the locations of the TRIUMPH ITF moorings (purple circles) and mixing measurements (blue star). The north entrance of the Lombok Strait is 1200-m deep and 35-km wide. The sill is 300-m deep and 22-km wide. Badung Sill is 105-m deep and 19-km wide. Alas Strait is 150-m deep and 21-km wide and Bali Strait is 70-m deep and 8-km wide. Gray lines are bathymetry contours. The red lines are the 80 milli^o^C fortnightly signal (MS_*f*_) sea surface temperature (SST) amplitude contour from (**c**). **f**, **g** Estimates of fortnightly (MS_*f*_) SST amplitude in milli^o^C during the southeast and northwest monsoon, respectively (Susanto and Ray 2022)

The Indonesian seas, with their complex geography and narrow passages, provide the only pathway for low-latitude Pacific Ocean water to flow into the Indian Ocean (Fig. 1a). Many previous studies have discussed the importance of Indonesian Throughflow (ITF) on water mass transformation, circulation, biogeochemistry, and heat/freshwater fluxes into the Indian Ocean (i.e., Bryden and Imawaki 2001; and reference thereafter in). The importance of tidal mixing for the transformation of Indonesian Throughflow waters and the climate system was reviewed by Sprintall et al. (2014). The detailed geography of nonlinear interactions between internal tides, tidally induced mixing and the influence of the ITF and strait geometry in the Indonesian seas are complicated and remain poorly understood to this day. Within the Indonesian seas, temperature and salinity stratification as well as sea surface temperature (SST) are significantly altered by turbulent mixing due to strong tidal forcing (Ffield and Gordon 1992; Ffield and Robertson 2005, 2008; Robertson 2010).

In situ observations show large water-mass modification occurring in the narrow southern passages where the flow exits the Indonesian archipelago (i.e., Atmadipoera et al. 2009). Temperature–salinity (TS) diagram from CTD measurements show a continual erosion of a salinity maximum centered around *σ*_*θ*_ = 24 kg m^−3^ from north to south through the Makassar and Lombok Straits (Fig. 1b). Between the CTD taken in the middle of Lombok Strait and one south of Lombok Strait the TS properties become nearly linear, with not only the salinity maximum, but also the deeper salinity minimum (centered on *σ*_*θ*_ = 26.5 kg m^−3^) having been mixed away. This abrupt change in water-mass properties indicates elevated mixing over the sills at the southern end of Lombok Strait. Remote sensing and numerical simulations strongly suggest that this mixing is tidally driven. Ray and Susanto (2016) found enhanced fortnightly satellite SST amplitudes localized to narrow straits and channels, including Lombok Strait with amplitudes of order 100 mK (0.1 °C) and ± 15% uncertainty. In situ observations using microstructure profiler confirm this finding (Nagai et al. 2021). Following Ffield and Gordon (1996), they suggested that the fortnightly cycle in SST is a result of mixing induced by internal tides. Using a numerical model that includes tides, Nugroho et al. (2018) show a similar map of fortnightly (MS_*f*_) amplitude. All these hotspots occur in regions that display strong semidiurnal (M_2_) current velocities (Ray et al. 2005), and where the model of Nagai and Hibiya (2015) predicts significant dissipation of baroclinic tidal energy.

Despite the fact that previous research has demonstrated the occurrences of intense mixing within the Indonesian seas and their effects on water mass transformation into the Indian Ocean, no comprehensive microstructure measurements have been conducted in the region with strong tidal mixing SST signatures. Prior microstructure measurements (Koch-Larrouy et al. 2015; Nagai et al. 2021) have focused on the spatial distributions of tidal mixing then the temporal variability. Alford et al. (1999) conducted the only exhaustive temporal microstructure measurements in the Banda Sea and concluded that the sea exhibits weak mixing. Using weekly and lower (1° × 1°) spatial resolution SST, Ffield and Gordon (1996) concluded that the Banda Sea indicates significant tidal mixing. Nonetheless, utilizing high (1 km × 1 km) spatial and daily temporal SST resolutions, Susanto and Ray (2022) and Ray and Susanto (2016) demonstrated that the Banda Sea exhibited weak mixing, which is consistent with in situ microstructure measurements (Alford et al. 1999). Field and Gordon used low temporal and spatial resolution of satellite-derived temperature data. Therefore, their conclusions on locations of strong mixing did not correspond to those of comprehensive microstructure measurements by Alford et al. (1999). Meanwhile, using high temporal and spatial resolution data, Ray and Susanto (2016; 2019) and Susanto and Ray (2022) showed that their conclusions on tidal mixing locations are consistent with the spatial distribution of tidal mixing as observed using microstructure profilers (Koch-Larrouy et al. 2015; Nagai et al. 2021). However, their microstructure measurements were not as comprehensive as those of Alford et al. (1999), in which Alford et al. continuously measured at the same location in the Banda Sea for two weeks. Therefore, it is essential to conduct comprehensive microstructure measurements in regions with strong SST tidal mixing signatures (Ray and Susanto 2016, 2019; Susanto and Ray 2022) for at least 2 weeks.

In this research, we present comprehensive in situ measurements south of the Lombok Strait to validate the strong mixing signatures observed from the SST (Susanto and Ray 2022; Ray and Susanto 2016, 2019). Susanto and Ray (2022) concluded that stronger tidal mixing during the southeast (boreal summer) monsoon and El Niño than that during the northwest (boreal winter monsoon) and La Niña. Therefore, the tidal mixing plays a critical role regulating seasonal and interannual variability of water mass transformation and Indonesian throughflow. In March 2021, we carried out microstructure measurements south of the Lombok Strait for consecutive 15 days with the aims to validate the mixing hotspots that significantly affect the SST and water mass transformations in one of the ITF exit passages into the Indian Ocean.

## Observations and method

Fifteen days of tidal mixing observations were collected from March 12 to 27, 2021 as part of the TRIUMPH mooring recovery and redeployment cruises. TRIUMPH is an international collaborative research among scientists from Indonesia led by the Indonesian Institute of Sciences (LIPI; and now becomes the National Research and Innovation Agency, BRIN) and the First Institute of Oceanography-MNR, China and University of Maryland, USA. This work was conducted onboard the Indonesian research vessel R/V Baruna Jaya VIII (operated and maintained by BRIN), the microstructure measurements were conducted in the south of the Lombok Strait with a radius of 0.05 degree and centered on 115.54^o^E and 9.02^o^S (blue star in Fig. 1e).

The goal was to obtain near-continuous measurements spanning a spring–neap cycle. Every three hours the MSS60 microstructure profiler (Sea & Sun Marine Technology) was deployed three times followed by CTD measurements using shipboard CTD. The free falling MSS60 microstructure profiler was equipped with a Seabird Instruments CTD, two shear air-foil probes, was ballasted to fall at 0.55 ms^−1^ and dropped from the starboard side of the vessel to a depth of 300 m. The shipboard RDI broadband 75-kHz ADCP provided 4-m resolution velocity profiles out to ~ 450 m. Retrieval of the MSS60 microstructure profiler was challenging due to strong shear/reversal flow between 100 and 300 m depth. Despite that, in all, we have successfully gathered 346 profiles of potential temperature, salinity and potential density, pressure, and kinetic energy dissipation rate during the 2 weeks. In addition, we have successfully gathered 111 CTD casts. The ship was also equipped with meteorological sensors to measure atmospheric parameters (wind speeds and directions, air temperature and pressure, humidity, and dew point).

The turbulent kinetic energy dissipation rate ($\varepsilon$) can be estimated from shear velocity measurements by air-foil probes (i.e., Gregg 1999; Lueck et al. 2002; Schafstall et al. 2010). Wave number spectra were calculated from 1 s of shear data (1024 individual measurements), which corresponds to a vertical interval of 0.5–0.6 m. Estimates of $\varepsilon$ are then derived by integrating the shear spectrum using the relationship for isotropic turbulence (Schafstall et al. 2010):

1 $$\varepsilon =7.5 \mu \overline{\left[{\left(\frac{\partial u}{\partial z}\right)}^{2}\right]}=7.5\mu \left({\int }_{{k}_{min}}^{{k}_{max}}{E}_{\frac{d{u}{\prime}}{dz}}\left(k\right)dk\right),$$

where $\mu$ is the dynamic viscosity of sea water, and ${E}_{du{\prime}/dz}$*(k)* is the shear wavenumber spectrum. The shear variance can be obtained by integrating the shear wavenumber spectrum from a lower wave number, *k*_*min*_ (taken as 3 cpm), to an upper cutoff number *k*_*max*_, which is depends on the Kolmogorov wavenumber (Nasmyth 1970; Moum et al. 1995; Prandke et al. 2000).

The processing routines largely followed best practice recommendations for analysis microstructure profiler (Lueck et al. 2024) and were tested against the benchmark data (Fer et al. 2024) recently published by the Scientific Committee on Oceanographic Research (SCOR) working group 160 “Analyzing ocean turbulent observations to quantify mixing” (ATOMIX). The dissipation rates determined by the routines used here were within a factor of square root 2 of the reference values when the $\varepsilon$ is smaller than 3–4 × 10^–6^ Wkg^−1^. We may need to remove shear data at higher *ε* values due to instrument vibration, which may not be removed by the process. Therefore, if necessary, manual correction was applied.

Calculation of $\varepsilon$ for each of the two shear sensors was done separately. Before both values are merged/averaged, the derived values for each probe are carefully edited for outliers/spikes resulting from biofouling and occasional jerking of the tether cable. The dissipation rates for the top 20 m are excluded due to possible contamination from the ship. To reassure the quality of the data, we selected data with quality spikeflag = 0 only. Following Osborn (1980) and Oakey (1982), turbulent eddy diffusivities ($K\rho$) are calculated from $\varepsilon$ and buoyancy frequency (*N*), and $K\rho =\Gamma \varepsilon /N$^*2*^*,* where mixing efficiency, $\Gamma$, set to 0.2.

## Results and discussion

**Fig. 2:**
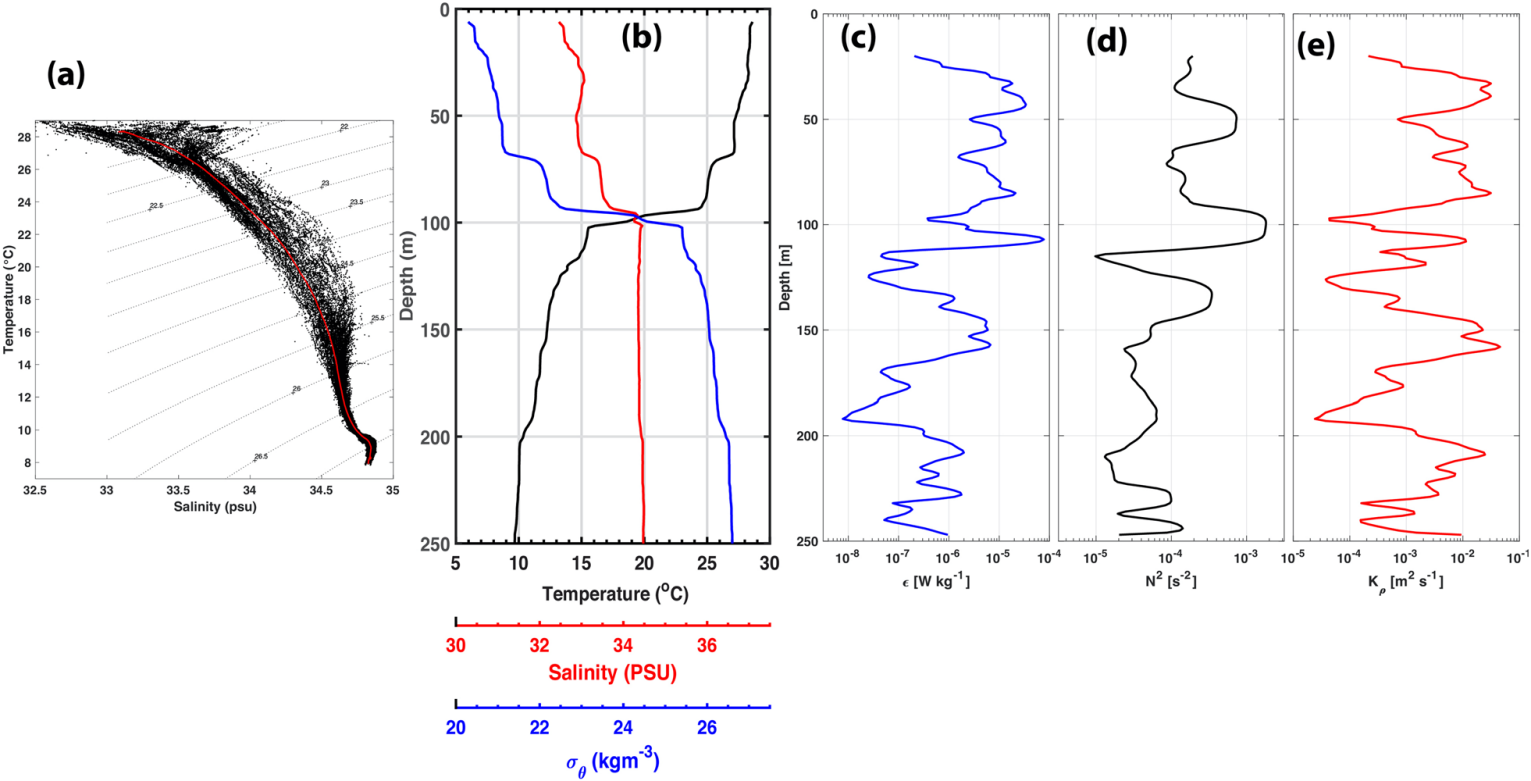
**a** Temperature–salinity profiles (91) taken during the mixing cruise. The red-line is the mean. **b** An example of typical CTD profile taken on March 23, 2021, at 03:00 am local time in the south of the Lombok Strait (Fig. 1). **c**–**e** The dissipation rate ($\varepsilon$), buoyancy frequency (N^2^), and diapycnal diffusivity ($K\rho$*)*, based on the MSS60 microstructure profiler taken subsequently after the shipboard CTD cast

Analysis of all CTD data taken during the 2-week cruise is shown in Fig. 2a. Strong variability of T–S diagram in the upper layer with potential density ~ 22.5 kg m^−3^, follows by the layer below with potential density 23.0 to 26.0 kg m^−3^. Meanwhile, Fig. 2b shows the typical depth profiles of temperature, salinity, and potential density. We can clearly see the extremely strong thermocline layer ~ 100 m with potential temperature and density changes up to 10 °C and 3 kg m^−3^, respectively. Figure 2c–e shows an example of the 1-m dissipation rate ($\varepsilon )$, N^2^, and $K\rho$ profiles taken on March 23 at 03:00 am local time. The strong thermocline layer is also manifested in the strong values of $\varepsilon$, N^2^, and $K\rho$, up to 10^–3^ Wkg^−1^, 10^–3^ s^−2^, and 10^–2^ m^2^s^−1^, respectively. The waving of this thermocline layer varies with the tides and manifested in the SST. Hence, the variability of SST due to tidal mixing can be extracted from SST analysis (Susanto and Ray 2022; Ray and Susanto 2016, 2019). Susanto and Ray (2022) suggested that the tidal mixing modulated by monsoon and interannual variability associated with Indian Ocean Dipole (IOD) and El Niño Southern Oscillation (ENSO). Stronger mixing occurs during the southeast monsoon (boreal summer) and El Niño phase than that during northwest monsoon and La Niña. Meanwhile, our mixing measurements conducted in the mid-March, which is a transition between northwest monsoon and southeast monsoon. Hence, it is expected that the high mixing values presented here may not the maximum mixing in south of the Lombok Strait. Additional observations are needed to validate this condition.

**Fig. 3:**
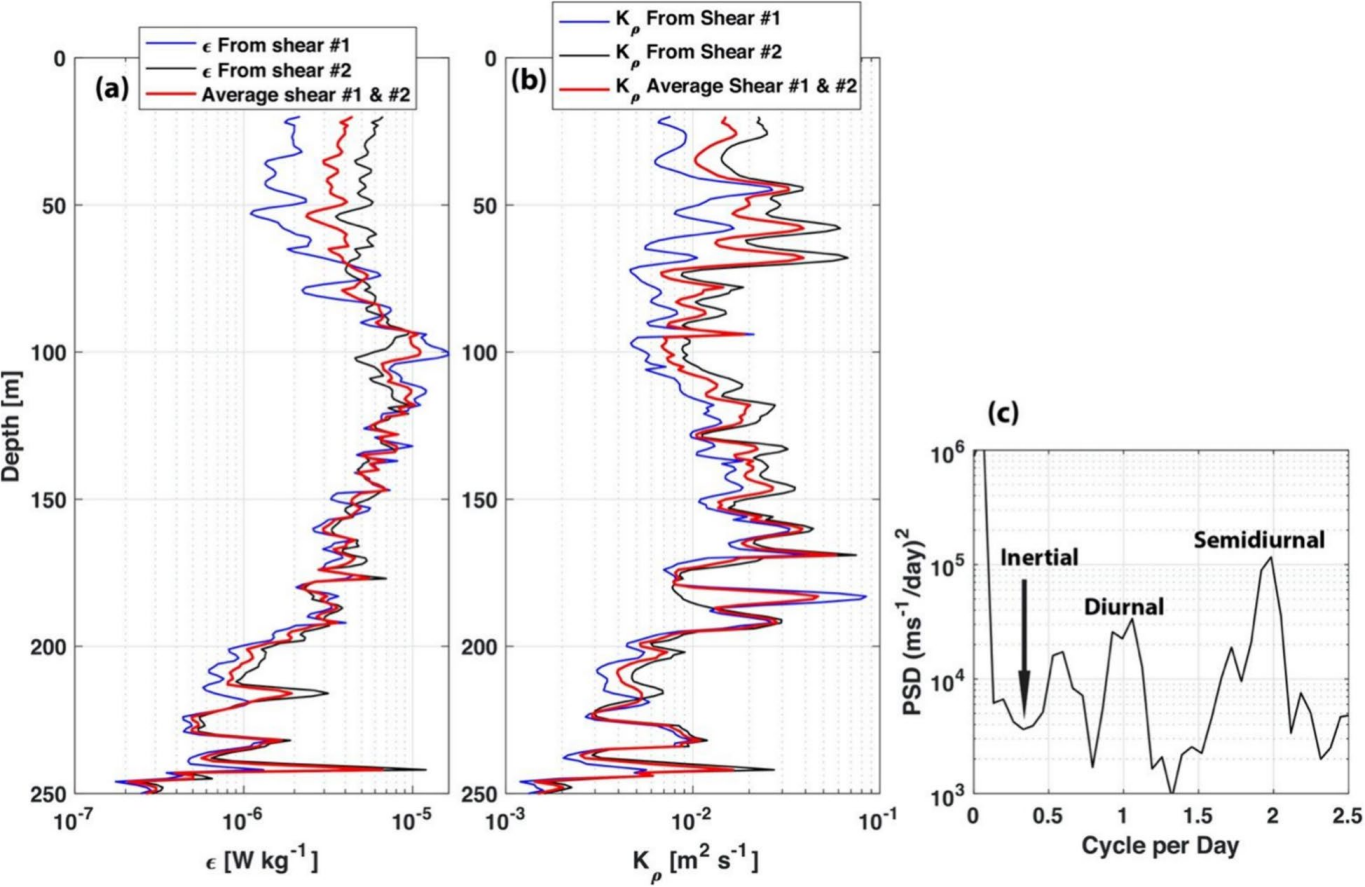
a Turbulent kinetic energy dissipation rate ($\varepsilon$) derived from shear sensor 1 and 2; and b diapycnal diffusivity ( $K\rho$ ) profiles derived from shear sensor #1 and 2; and c power spectrum density of the flow (averaged from 100 to 200 m) derived from the onboard ADCP during the 2-week cruise

To further quantify the turbulent kinetic energy dissipation rate ($\varepsilon$) and diapycnal diffusivity ($K\rho$) profiles during the 2-week cruise, we calculated their temporal means (Fig. 3) and their depth averaged from 20 to 250 m as well as their associated standard deviation (Table 1). In general, the dissipation rate ($\varepsilon$) and diapycnal diffusivity ( $K\rho$) profiles from both shear sensors are in synch. In addition, the spectrum of the flow (averaged for 100 m to 200 m where we observed strong isotherm displacement and shear) during the cruise is presented in Fig. 3c. The temporal and depth averaged of the turbulent kinetic energy dissipation rate and the diapycnal diffusivity from 20 to 250 m are $\varepsilon$ = (4.15 ± 15.9) × 10^–6^ W kg^–1^ and $K\rho$ = (1.44 ± 10.7) × 10^–2^ m^2^s^–1^, respectively. As a comparison, the temporal and depth averaged of diapycnal diffusivity for the Banda Sea is $K\rho$ = (9.2 ± 0.55) × 10^–6^ m^2^s^–1^ (Alford et al. 1999), and the “open ocean” within 2° of the equator to at 50°–70° is $K\rho$ = 0.03 × 10^–4^ m^2^s^−1^ to (0.4–0.5) × 10^–4^ m^2^s^−1^ (Kunze et al. 2006). Therefore, the south Lombok Strait’s diapycnal diffusivity $K\rho$ is 10^4^ times greater than the Banda Sea. These results also confirmed previous microstructure measurements by Nagai et al. (2021). These are significant results that show that indeed, high-resolution satellite-derived sea surface temperatures can be used to narrow down the locations of strong tidal mixing. Hence, we can apply a similar technique to the world ocean; even in regions that are difficult and logistically challenging to carry out oceanographic cruises (i.e., due to piracy).

**Table 1:** The temporal mean (15 days) and depth averaged (20m - 250m) of turbulent kinetic energy dissipation rate ($\varepsilon$) and diapycnal diffusivity ($K\rho$) from two shear sensors of MSS60 microstructure profiler

	Turbulent kinetic energy dissipation rate ($\varepsilon$)	Diapycnal diffusivity ($K\rho$)
Shear #1	(3.83 ± 17.8) × 10^–6^ W kg^–1^	(1.09 ± 8.2) × 10^–2^ m^2^s^–1^
Shear #2	(4.42 ± 17.3) × 10^–6^ W kg^–1^	(1.79 ± 13.8) × 10^–2^ m^2^s^–1^
Average	(4.15 ± 15.9) × 10^–6^ W kg^–1^	(1.44 ± 10.7) × 10^–2^ m^2^s^–1^

**Fig. 4:**
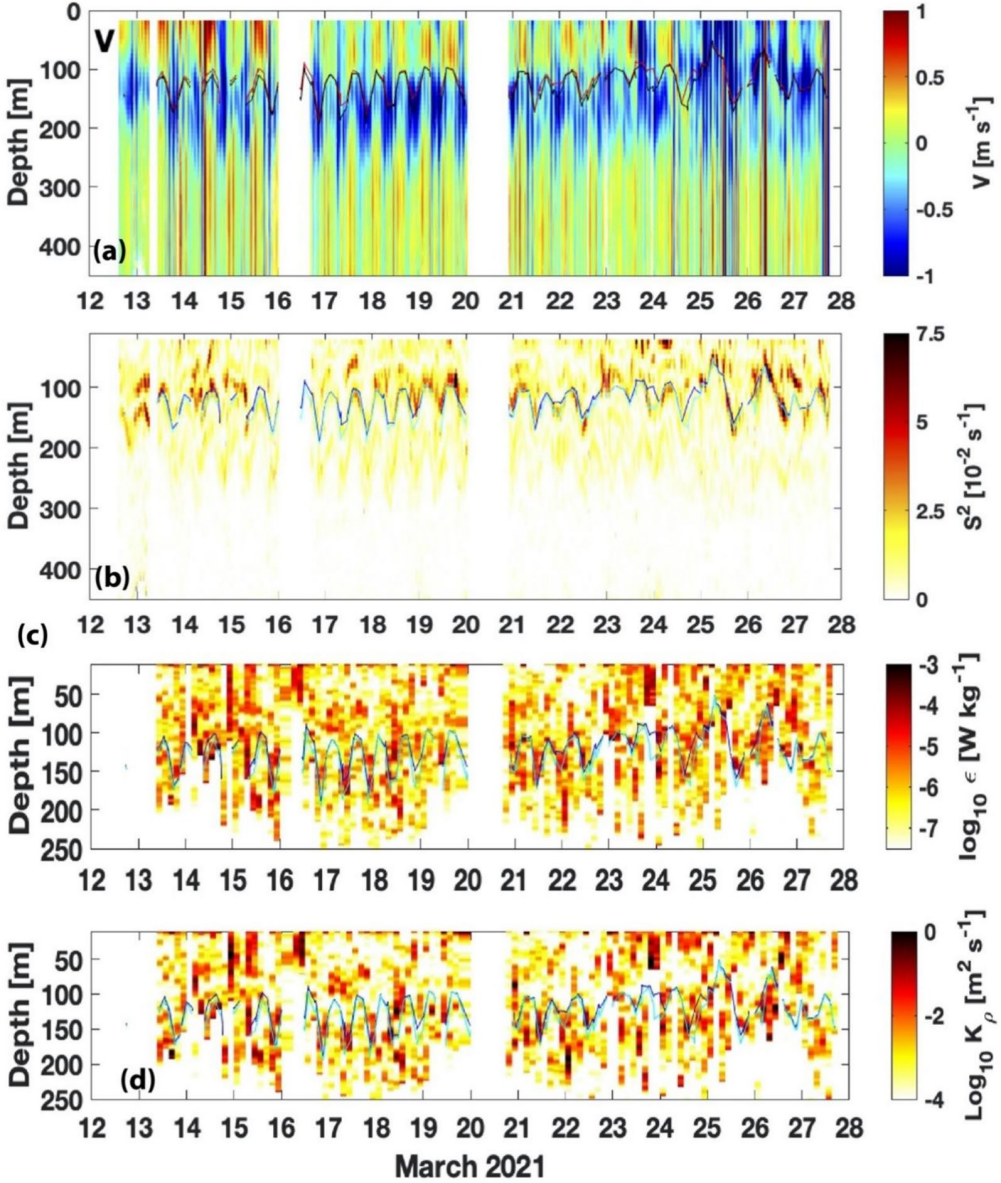
**a** Meridional velocity (V) derived from shipboard ADCP during the mixing cruise in south of the Lombok Strait from March 12–27, 2021, and overlaid with 18 °C isotherm (red) and 25 kg m^−3^ isopycnal (black). **b** Vertical shear squared derived from the zonal and meridional velocities of shipboard ADCP. **c** Turbulent kinetic energy dissipation rate ($\varepsilon$) and **d** diapycnal diffusivity ( $K\rho$ profiles during the 2-week cruise, overlaid with 18 °C isotherm (blue) and 25 kg m^−3^ isopycnal (cyan)

In addition to analyzing the free-fall microstructure profiles, we analyzed velocity profiles based on the onboard ADCP. Time series of north–south velocity, velocity shear, dissipation rate, and diapycnal diffusivity are shown in Fig. 4. During the period of the observations the ITF was typically centered around 150 to 200 m depth. The upper 450 m of the water column (all that was measured) was strongly influenced by semidiurnal tides. Above and below the southward flowing ITF, the currents are often northward. Around March 25 there is a prolonged period of southward flow at the surface. The upper interface of the ITF is generally well described by either the 18 °C isotherm or the 25 kg m^−3^ isopycnal, and had semidiurnal vertical displacements of O(50 m) (Fig. 4a). The largest shears and dissipation rates tend to be on this upper interface of the ITF, with little shear observed at the base of the ITF (Fig. 4b). Figure 4c and d shows the variability of turbulent kinetic energy dissipation rate ($\varepsilon$) and turbulent eddy diffusivities ($K\rho$) that vary with tides.

Figure 4a shows isotherm displacement, which indicates the presence of an internal wave (Tai et al. 2020; Morozov et al. 2019; Lim and Park 2022), as depicted by Fig. 1d (white lines, i.e., Susanto et al. 2005; Matthews et al. 2011; Purwandana et al. 2021), which can induce velocity shear and drive mixing (Masunaga et al. 2016; Kawasaki et al. 2021). One way to detect and measure internal tides is by observing the vertical displacement of the isotherms. The amplitude of the internal tides is related to the magnitude of the vertical displacement of isotherms. Based on the isotherm displacements in Fig. 4a, the internal tide amplitude in the south of the Lombok Strait is up to 100 m with a mean of 55 m, which is slightly lower than their companion, the northward propagation of internal waves, which may reach more than 100 m in amplitude (Susanto et al. 2005).

Figure 3c indicates that tides induce these internal waves, with the semidiurnal tide being more dominant than the diurnal tide. The inertial frequency for the region (9^o^S) is 0.31 cycles per day which is neither diurnal nor semidiurnal. To further quantify the relation between the velocity flow and the turbulent kinetic energy dissipation rate ($\varepsilon$) and diapycnal diffusivity ($K\rho )$ profiles during the 2-week cruise, we calculated the correlation between them at 150 m depth, where we observed strong isotherm displacement and shear. The correlation coefficients for V150 and dissipation rate (*ε*) (from shear probes 1 and 2) are 0.78 and 0.79. V150 and diapycnal diffusivity (Kρ) (from shear probes 1 and 2) have a correlation of 0.77 and 0.76, respectively. These correlation coefficients are above the 95% significance level. There is a semidiurnal frequency coherence between V150, dissipation rate, and diapycnal diffusivity. This indicates the influence of tides on the mixing.

The velocity shear is caused by the difference in the speed of the internal tides between the surface (upper layer) and lower layer (seafloor), which creates a horizontal gradient in the velocity field. This gradient can lead to the generation of turbulence, which in turn can cause the mixing of water masses (Masunaga et al. 2016; Fang et al. 2023). While the exact mechanism of internal tide-induced mixing is still being studied, it is believed that the turbulence generated by velocity shear can cause the mixing of water masses with different properties, such as temperature and salinity. In the case of the Lombok Strait, the strong vertical shear of the intensified ITF from the Pacific and semidiurnal tides from the Indian Ocean obstructed by the Lombok’s Sill at the southern end of the Strait (350 m) are ideal places for internal tide generation, propagation, and tidal mixing hotspots.

## Conclusions

Our comprehensive field measurements of tidal mixing using a free-fall microstructure profiler in the south of the Lombok Straits, Indonesia, for 15 consecutive days validate the previous remotely sensed approach on strong tidal mixing locations (Ray and Susanto 2016, 2019). The temporal and depth averaged of the diapycnal diffusivity ($K\rho$ )is (1.44 ± 10.7) × 10^–2^ m^2^ s^–1^, which is up to 10^4^ folds greater than the Banda Sea is (9.2 ± 0.55) × 10^–6^ m^2^ s^–1^ (Alford et al. 1999) or “open ocean” values (Kunze et al. 2006). These results in the south of the Lombok Strait open up the possibility of remote sensing sea surface temperature data to estimate tidal mixing in other regions around the global ocean, even in the regions that are difficult to carry out direct in situ observations due to various reasons such as logistics and security. Given the strong tidal mixing located along the exit passages of the Indonesian seas to the Indian Ocean, interactions between internal tide-induced mixing and ITF have strong implications for water mass transformations feeding to the Indian Ocean, which may have implications for water circulation and climate.

Our comprehensive mixing measurements conducted in south of the Lombok Strait in the mid-March 2021, which is a transition between northwest monsoon and southeast monsoon. Susanto and Ray (2022) suggested that the tidal mixing modulated by monsoon and interannual variability associated with Indian Ocean Dipole (IOD) and El Niño Southern Oscillation (ENSO). Stronger mixing occurs during the southeast monsoon (boreal summer) and El Niño phase than that during northwest monsoon and La Niña. Hence, even though the diapycnal diffusivity in the south of the Lombok Strait is up to 10^4^ folds greater than that in the open ocean, it is expected that the high mixing values presented here may not be the maximum mixing in the south of the Lombok Strait. Validation of this condition requires additional observations. In addition, the exact mechanism of how internal tides induce mixing and why there is a north–south asymmetric feature of tidal mixing signatures are still being investigated. The relationship between the vertical displacement of isotherms and internal tides requires further study to understand the complex interactions between these phenomena.

### Supplementary Information

Supplementary Material 1: Shear power spectra (solid blue line) of shear 1 (a) and shear 2 (b) for MSS profile taken on March 23, 2021, at 03:00am local time (Figure 2). We select 100m depth in which we observed strong thermocline layer where dissipation rate for shear 1 and shear 2 have large differences (Figure 3). The Nasmuth spectrum is shown in dash-black line. However, as the MSS uses high-pass filter to filter the shear sensor data, and as the shear sensor tip is about 8-mm long and has a bullet shape, the spectra of the shear sensor biased low at low wave numbers and at high wave numbers. Instead correcting the shear spectra for the lost shear variance due to the low-pass and finite sensor tip, we correct the Nasmuth spectra. The Nasmuth spectrum, that is fitted to the shear spectrum level within k_min and k_max (red vertical lines) is displayed as thick black solid line surrounded by two yellow lines.

## Data Availability

The Group for High-Resolution Sea Surface Temperature (GHRSST) daily blended SST data were produced by the Remote Sensing System and obtained from the NASA EOSDIS Physical Oceanography Distributed Active Archive Center at the Jet Propulsion Laboratory, Pasadena, CA. This data is publicly available from NASA-JPL-PODAAC website: https://podaac.jpl.nasa.gov/dataset/MW_IR_OI-REMSS-L4-GLOB-v5.0. We are currently working with the Digital Repository University of Maryland (DRUM; https://drum.lib.umd.edu/home) to deposit the in situ data and have a DOI that will be active soon. However, the data is available upon request. DRUM is a long-term, open-access repository managed and maintained by the University of Maryland Libraries.
