# Supplementary material for: Field measurements of turbulent mixing south of the Lombok Strait, Indonesia
Source: Geosci Lett. 2024 Aug 14;11(1):36. doi: 10.1186/s40562-024-00349-3 (PMC11324699; doi:10.1186/s40562-024-00349-3)
Supplement: Supplementary file 1 — Supplementary Material 1: Shear power spectra (solid blue line) of shear 1 (a) and shear 2 (b) for MSS profile taken on March 23, 2021, at 03:00am local time (Figure 2). We select 100m depth in which we observed strong thermocline layer where dissipation rate for shear 1 and shear 2 have large differences (Figure 3). The Nasmuth spectrum is shown in dash-black line. However, as the MSS uses high-pass filter to filter the shear sensor data, and as the shear sensor tip is about 8-mm long and has a bullet shape, the spectra of the shear sensor biased low at low wave numbers and at high wave numbers. Instead correcting the shear spectra for the lost shear variance due to the low-pass and finite sensor tip, we correct the Nasmuth spectra. The Nasmuth spectrum, that is fitted to the shear spectrum level within k_min and k_max (red vertical lines) is displayed as thick black solid line surrounded by two yellow lines. [file 40562_2024_349_MOESM1_ESM.docx]

**Supplement Figure**


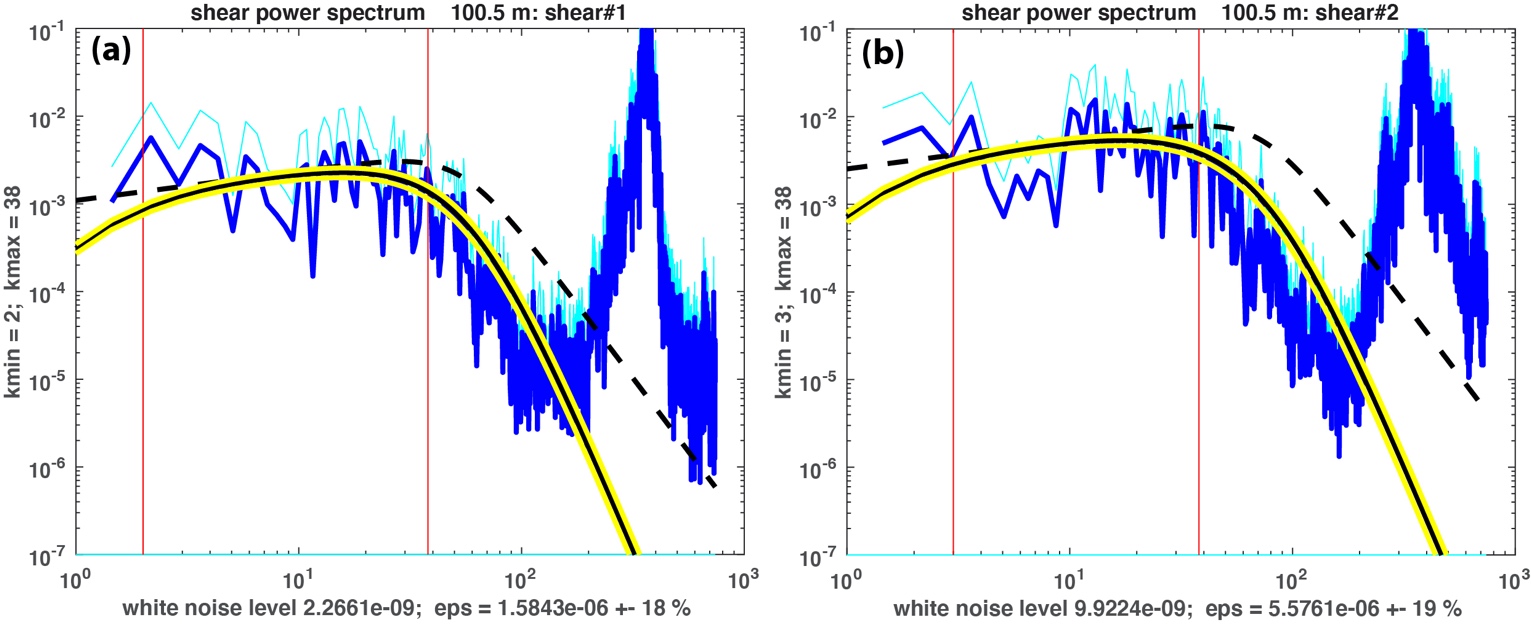


**Supplement Figure.** Shear power spectra (solid blue line) of shear 1 (a) and shear 2 (b) for MSS profile taken on March 23, 2021, at 03:00am local time (Figure 2). We select 100m depth in which we observed strong thermocline layer where dissipation rate for shear 1 and shear 2 have large differences (Figure 3). The Nasmuth spectrum is shown in dash-black line. However, as the MSS uses high-pass filter to filter the shear sensor data, and as the shear sensor tip is about 8-mm long and has a bullet shape, the spectra of the shear sensor biased low at low wave numbers and at high wave numbers. Instead correcting the shear spectra for the lost shear variance due to the low-pass and finite sensor tip, we correct the Nasmuth spectra. The Nasmuth spectrum, that is fitted to the shear spectrum level within k_min and k_max (red vertical lines) is displayed as thick black solid line surrounded by two yellow lines.
